# Childhood Trauma, Cognition, and Eating Psychopathology: A Network Analysis

**DOI:** 10.3390/healthcare13060630

**Published:** 2025-03-14

**Authors:** Kathryn Pasquariello, David A. Gansler, Sukanya Ray, Malvina O. Pietrzykowski, Margaret Pulsifer, Christina Ralph-Nearman

**Affiliations:** 1 Department of Psychology, Suffolk University, Boston, MA 02108, USA; 2 Department of Psychiatry, Harvard Medical School, Massachusetts General Hospital, Boston, MA 02114, USA; 3 Department of Psychological and Brain Sciences, University of Louisville, Louisville, KY 40292, USA

**Keywords:** eating disorders, cognition, childhood trauma, self-concept, network analysis

## Abstract

**Background/Objectives**: Childhood trauma is associated with psychiatric sequelae beyond post-traumatic stress disorder (PTSD), including eating disorders (EDs) and cognitive dysfunction. While eating pathology is related to cognition irrespective of childhood trauma exposure, such experiences may influence the way in which these symptoms develop. One method that has garnered increased interest in studying the interrelationships between symptoms and pinpointing core features of psychopathology is network analysis. **Methods**: Using data from the Nathan Kline Institute Rockland Sample, the present study utilized network analysis to examine associations between ED symptoms and cognitive deficits among a community sample. Comorbidity networks were constructed in two samples: adult reporters of childhood trauma (*n* = 116) and non-reporters (*n* = 101). **Results**: In line with the cognitive-behavioral model of EDs, overvaluation of weight/shape was central to both networks but demonstrated higher strength centrality among trauma reporters. Additionally, among trauma reporters, executive functioning deficits were linked to food-related attentional biases; alternatively, affective symptoms were salient among non-reporters. Finally, negative self-concept (theorized as a putative consequence of cognitive deficits) was implicated in both networks. When comparing the networks according to global strength, we did not find significant differences. **Conclusions**: Our findings contribute to the literature examining the interrelatedness of eating pathology and cognition and extend these findings by considering the role of trauma exposure. While our networks shared features of overvaluation of weight/shape and negative self-concept, they differed according to cognitive-affective concomitants. This information holds clinical utility in advancing assessment and intervention for individuals with eating psychopathology.

## 1. Introduction

Research suggests that childhood trauma represents a powerful antecedent of eating psychopathology in adulthood [1]. Moreover, the prevalence of comorbid post-traumatic stress disorder (PTSD) in patients with an eating disorder (ED) is estimated to range from 9 to 24%, with research suggesting that comorbid PTSD is associated with more severe ED psychopathology [2,3,4].

Childhood trauma has also been linked to cognitive difficulties in adulthood. Retrospective data among adults with attention-deficit/hyperactivity disorder (ADHD) indicate high prevalence rates of childhood abuse [5]. Further, a study conducted by Brown and colleagues [6] demonstrated that children with a parent-reported ADHD diagnosis had a significantly higher prevalence of adverse childhood experiences (ACEs). In a meta-analysis conducted by Op den Kelder et al. [7], trauma-exposed youth were shown to perform worse on executive functioning tasks, presumably due to the neurobiological effects of trauma exposure on key brain regions associated with these tasks. From a clinical perspective, it is thought that avoidance symptoms resulting from traumatic experiences can cause distractibility, which mirrors ADHD symptoms [8]. Taken together, early trauma exposure may confer risk beyond the development of PTSD, including increased risk of both maladaptive eating-related behaviors and cognitive difficulties.

One pathway through which childhood trauma exposure may be related both to maladaptive eating patterns and cognitive problems is the sense of self, a characteristic on which trauma exposure can exert profound and lasting impacts [9]. Research suggests that among individuals with anorexia nervosa (AN), for example, difficulties forming a consistent sense of self are associated with the maintenance of ED symptoms [10]. Further, negative self-evaluation has been shown to mediate the relationship between early trauma exposure and overvaluation of weight/shape among individuals with binge eating disorder (BED; [11]). Poor self-concept has also been suggested to be a downstream consequence of ADHD behaviors [12]. As such, poor self-concept may be a shared feature of eating pathology and cognitive deficits, irrespective of trauma exposure. However, early traumatic experiences may be relevant to the presentation of these symptoms as they arise in adulthood, revealing key differences in mechanisms that maintain co-occurrence.

To date, the way in which relationships between cognitive deficits and eating behaviors are interrelated in reporters versus non-reporters of childhood trauma is unclear. Clarifying how these functional pathways differ according to early trauma exposure could inform intervention planning for individuals who are suffering from related psychopathology. Moreover, considering the heterogeneity in symptom trajectories associated with post-traumatic stress experiences and eating pathology, it is important to identify “key” symptoms that may be shared across individuals. This information can then be used to inform evidence-based treatments by suggesting additional psychological targets. One method that has been used in the last decade to explain the interconnectivity and relative importance of symptom-level relationships between a given condition or comorbid conditions—and depict these relationships through a dynamic modeling process—is network analysis.

While traditional conceptualizations of psychopathology presume that clinical symptoms are reflective of an underlying common cause, network theory adopts a causal systems perspective, suggesting that symptoms are constitutive, rather than reflective, of mental illness [13]. According to network theory, symptoms, represented as “nodes”, are part of a dynamic, reciprocal network that produces, sustains, and underlies psychological conditions [14,15]. Nodes are connected via “edges” to form a web-like model of psychopathology. Network analysis has the ability to directly test chains of symptoms that have been postulated in theoretical models, such as the role of overvaluation of weight and shape that is outlined in the cognitive-behavioral model of EDs [16].

The present research aims to build upon existing network analyses of post-traumatic stress and disordered eating (e.g., [17]) by examining how early trauma exposure influences eating problems (i.e., EPs) and cognitive problems typical of ADHD (i.e., CPs) in a community sample of adults. The analyses in this study include (a) an examination of node centrality among adults who reported at least one traumatic event during childhood and (b) a comparison of symptom interactions between EPs and CPs among reporters (i.e., T+) versus non-reporters (i.e., T−) of childhood trauma, to better understand how network structures differ between samples. As such, the present study considers childhood trauma exposure to be an upstream vulnerability factor for downstream consequences (e.g., [18,19]) related to adult EPs and CPs while also accounting for unique relationships between these constructs, irrespective of trauma reporting status.

In line with the cognitive-behavioral model of EDs [16], we expected to find that EPs related to overvaluation of weight and shape would be central to both networks but hypothesized these symptoms to be more salient to the T+ network. Given the literature to suggest that sense of self is implicated both in traumatic experiences and in our symptom constructs of interest, we also proposed that negative beliefs and attitudes related to the self (as a secondary consequence of cognitive challenges) would be a central feature of both networks, but again exhibit stronger centrality in the T+ network. Finally, given the empirical support for a relationship between early trauma exposure and cognitive functioning, we expected to find that symptoms characteristic of executive deficits would be salient to the T+ but not the T− network.

## 2. Materials and Methods

### 2.1. Participants and Procedures

Anonymized self-report data for this study (collected between 2011 and 2022) were obtained from the Nathan Kline Institute Rockland Sample (NKI-RS; [20]). The NKI-RS is a nationally representative community sample of individuals aged 6–85 years old from Rockland County, New York, that aims to study the causes, treatment, prevention, and rehabilitation of mental illnesses. Zip code–based recruitment was conducted through mail, print advertising, and electronic advertising. The current study utilized a cross-sectional adult subset of individuals aged 16–85 years old, for whom written informed consent was obtained prior to undergoing a comprehensive assessment with trained personnel during a 1- or 2-day visit to the NKI. Participants were remunerated $200 to $250 plus travel and meal costs.

Participants were included in the present study if they indicated the ability to read and write fluently in English and had full self-report data regarding childhood trauma exposure, eating disorder assessment, and cognitive symptom assessment. The sample was then stratified according to the following cutoff criteria: participants had to be aged between 16 and 65 years old, had to have obtained a score of at least 1.7 on a global measure of eating disorder severity (i.e., sub-clinical; [21,22]), and had to have a T-score of 60 or above (i.e., elevated; [23]) on measures of behavioral ADHD indices. Additionally, we excluded participants who met the criteria for psychotic disorders and substance use disorders. From an initial sample of 1025 participants, 217 met our selection criteria. The demographic information of this resulting, final sample, stratified by trauma history, is presented in Table 1.

### 2.2. Instruments

#### 2.2.1. Childhood Trauma Exposure

The UCLA Child/Adolescent PTSD Reaction Index for DSM-IV (PTSD-RI; [24]) is a 48-item self-report measure that screens for exposure to traumatic events and related DSM-IV PTSD psychopathology in school-age children and adolescents. Though indicated for children and adolescents, the NKI-RS research team administered this assessment to all participants; this approach is supported by research to suggest that the measure can be reliably used with adult populations [25]. For the purposes of this study, we focused on Questions 1–13, which ask the participant, in a dichotomous format, whether they have experienced a DSM-IV Criterion A trauma [26]. Participants were assigned to the T+ sample if they responded “*yes*” (1) to experiencing any of the 13 traumatic events (*n* = 116) and were assigned to the T− sample if they did not endorse any such experience (*n* = 101).

#### 2.2.2. Eating Problems

The Eating Disorder Examination Questionnaire (EDE-Q 6.0; [27]) was used to assess EPs. The EDE-Q is a 28-item self-report measure adapted from the semi-structured clinical interview, the Eating Disorder Examination [28]. It is used to assess dietary restraint, eating concern, shape concern, and weight concern and evaluate the frequency of ED behaviors. This instrument contains variables that automatically calculate the participant’s score on the EDE-Q’s four subscales (restraint, eating concern, shape concern, and weight concern) and their global score. A global score of 2.3 is generally regarded as the cutoff for clinical ED symptoms [29]; while our criteria fell slightly below this cutoff, we chose a score of 1.7 in accordance with Nagata et al. [22] as it allowed us to balance power considerations with capturing clinical symptomatology. The EDE-Q has demonstrated inter-rater reliability coefficients between 0.91 and 1.0 for scores on the behavior frequency items, and test–retest reliability for these items ranges from 0.70 to 0.97 [30]. Cronbach’s α in our sample was 0.88.

#### 2.2.3. Cognitive Problems

The Conners’ Adult ADHD Rating Scales—Self Report: Short Version [23] was used to assess CPs. The CAARS-S:S is a 26-item self-report measure that is designed to assess current ADHD symptoms in adults. Participants rate the frequency of specific behaviors and problems associated with ADHD (i.e., trouble with organization, interrupting others) on a 4-point Likert scale ranging from 0 (*not at all*) to 3 (*very frequently*). These responses are factored into four subscales (Inattention, Hyperactivity, Impulsivity, and Problems with Self-Concept), which together yield an ADHD Index T-score. Scores on these subscales can range from 30 to 90+ (*M* = 50); a score above 60 is considered elevated across all four subscales. The CAARS-S:S has demonstrated good internal consistency (i.e., >0.88 [31]) and test–retest reliability (0.89; [32,33]) among community and clinical populations. Cronbach’s α in our sample was 0.90.

### 2.3. Statistical Analysis

#### 2.3.1. Manipulation Check

Given our approach of dividing the participant pool into T+ and T− samples based on a checklist response, we first conducted one-tailed, independent samples *t*-tests on the full sample from which our data was drawn (N = 1025) as a manipulation check. This sample was not restricted to differential index scores on the EDE-Q or the CAARS but instead included all participants who had self-report data on trauma history, EDE-Q, and CAARS-S:S. Our initial sample was predominantly White (76%), cisgender women (63%), with a mean age of 47 years old (*SD* = 18.5). Reporters of trauma in our larger sample scored significantly higher on the EDE-Q Global Score (*M* = 1.4, *SD* = 1.1, *n* = 683) compared to non-reporters (*M* = 1.1, *SD* = 1.0, *n* = 342); *t*(1023) = 2.8, *p* = 0.003, one-tailed. Trauma reporters also scored significantly higher on the CAARS-S:S ADHD Index (*M* = 47.1, *SD* = 8.1, *n* = 683) compared to non-reporters (*M* = 45.3, *SD* = 8.5, *n* = 342); *t*(1023) = 1.9, *p* = 0.02, one-tailed. These results suggest that meaningfully different samples can be created based on differential checklist responses.

#### 2.3.2. Network Estimation

We constructed two cross-sectional regularized partial correlation network models using R-4.2.2 studio. The networks were estimated using the Graphical Gaussian Model (GGM; [34]) approach and were regularized using the least absolute shrinkage and selection operator (LASSO; [35]). When applied to the edge weights of a network, this method shrinks small and potentially spurious relationships to exactly zero, creating a more parsimonious network [36].

#### 2.3.3. Network Visualization

Networks were visualized using the *qgraph* R package (R-4.2.2 studio) [37]. Networks consist of two elements: nodes and edges. Within the graphical network, each node depicts an individual symptom or problem, and each edge depicts a regularized partial correlation between two symptom nodes [15]. Positive edges are represented by solid, purple lines, while negative edges are represented by dashed, red lines. Line thickness reflects the strength of the association, whereby thicker edges represent stronger associations. The layout of the models is based on the *Fruchterman–Reingold* algorithm [38], which optimizes the topological presentation of nodes according to shared relationships. The nodes in our networks (11 EP nodes extracted from the EDE-Q and 5 CP nodes extracted from the CAARS-S:S) were determined via theoretical knowledge about cognitive-behavioral models of EDs and statistical process to assess for overlapping constructs, thus reducing redundant nodes (i.e., *goldbricker* function in R). A full list of the nodes and their corresponding item question is available in Supplementary Materials.

#### 2.3.4. Network Centrality

Measures of centrality were extracted from each network to characterize the importance of specific relationships and nodes in the networks. These indices included strength, expected influence (EI), betweenness, and closeness. While strength and EI refer to the sum of edge weights connected to a node, betweenness and closeness refer to the distance between nodes in a network. In the network approach to psychopathology, expected centrality influence rests on the assumption that deactivating highly ‘central’ nodes would proliferate to other nodes in the network, thereby collapsing the overall network structure and alleviating symptoms. While there are numerous graph theoretical measures that can be used for calculating centrality, these metrics are consistent with the prior network analysis literature [13].

#### 2.3.5. Network Stability

The accuracy and stability of network centralities were assessed using the bootstrap approach in the *bootnet* package in R [39] at 95% confidence intervals. To gain a stable and interpretable centrality, it is recommended that the correlation stability (CS) coefficient obtained from these analyses be at least 0.25 [15], which reflects the maximum number of cases that can be dropped to maintain a correlation between original centrality indices and those from subsamples that is 0.70 or higher.

#### 2.3.6. Network Comparison

To test for differences between reporters and non-reporters of childhood trauma, the networks were compared according to global strength [*S*], which represents the difference between the sum of the strengths of all edge weights in a network. This comparison was done via a network comparison test (NCT) using the R package *NetworkComparisonTest* 2.0.1. [40].

## 3. Results

### 3.1. Network Stability and Accuracy

Network stability and accuracy analyses are available in Supplementary Materials. The strength centrality for the T+ and T− networks had an acceptable level of stability (i.e., CS-coefficient = 0.30; 0.29, respectively). The centrality of EI also had an acceptable level of stability (i.e., CS-coefficient = 0.30; 0.25). The betweenness and closeness centrality fell below the suggested cutoff for stability (i.e., betweenness CS-coefficient = 0.05; 0.052, respectively, closeness CS-coefficient = 0.164; 0.086). This is consistent with prior findings that strength has always been the most accurately estimated centrality metric in psychopathology networks [39] (Epskamp et al., 2017). Given that betweenness and closeness did not result in stable parameter estimates, we chose not to interpret these centrality indices in the remainder of the manuscript.

### 3.2. Network Centrality

#### 3.2.1. Reporters of Childhood Trauma

**Figure 1:**
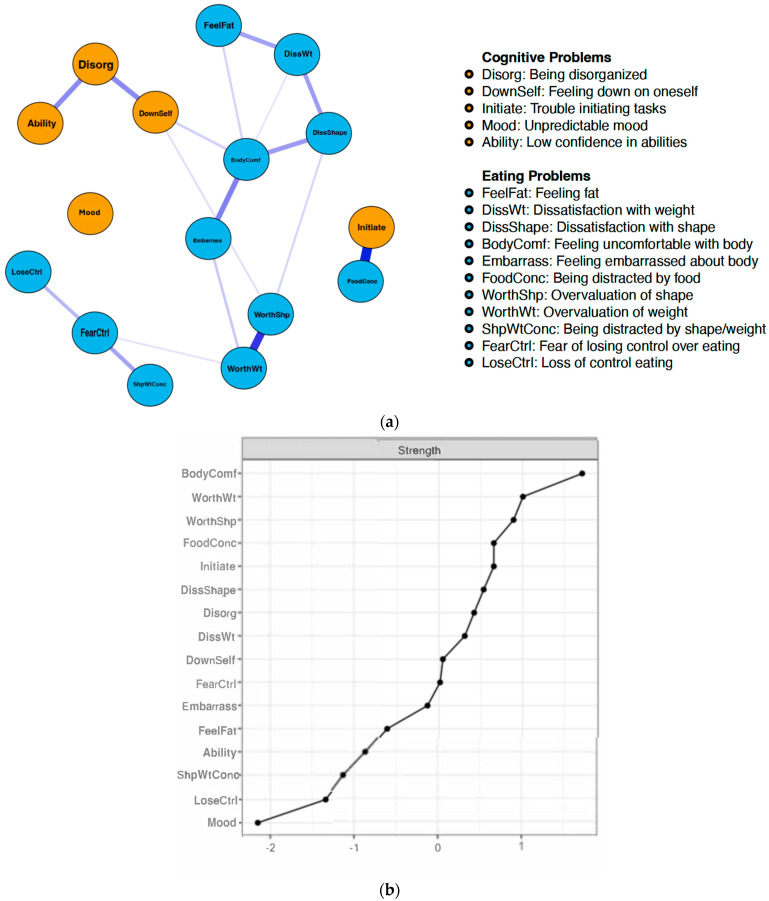
(**a**). Network structure of reporters of trauma. Blue circles (i.e., nodes) represent eating disorder symptoms (i.e., EDE-Q), and orange circles represent cognitive symptoms (i.e., CAARS-S:S). Thicker lines between nodes represent stronger associations. (**b**). Strength centrality plot of eating problems and cognitive problems in the network of reporters of trauma. Strength indices are standardized (*M* = 0, *SD* = ±1), where higher scores indicate greater centrality. BodyComf = Feeling uncomfortable with body; WorthWt = Overvaluation of weight; WorthShp = Overvaluation of shape; FoodConc = Being distracted by food; Initiate = Trouble initiating tasks; DissShape = Dissatisfaction with shape; Disorg = Being disorganized; DissWt = Dissatisfaction with weight; DownSelf = Feeling down on oneself; FearCtrl = Fear of losing control over eating; Embarrass = Feeling embarrassed about body; FeelFat = Feeling Fat; Ability = Low confidence in abilities; ShpWtConc = Being distracted by shape/weight; LoseCtrl = Loss of control eating; Mood = Unpredictable mood).

Figure 1a,b depicts the network structure and strength centrality of the T+ sample. The CP nodes with the greatest strength centrality among reporters of childhood trauma were trouble initiating tasks (*strength* = 0.61), being disorganized (*strength* = 0.43), and feeling down on oneself (*strength* = 0.10). The EP nodes with the greatest strength centrality in this network were discomfort with one’s body (*strength* = 1.55), overvaluation of weight (*strength* = 1.0), and overvaluation of shape (*strength* = 0.97). In this network, symptoms clustered mostly according to disorder (i.e., ADHD versus ED), with the exception of a relationship between being distracted by food/eating (EP; *strength* = 0.61) and trouble initiating tasks (CP; *strength* = 0.61). In addition to this association, strong positive connections emerged between the overvaluation of weight and the overvaluation of shape (EPs), as well as being disorganized and having low self-concept (CPs).

#### 3.2.2. Non-Reporters of Childhood Trauma

**Figure 2:**
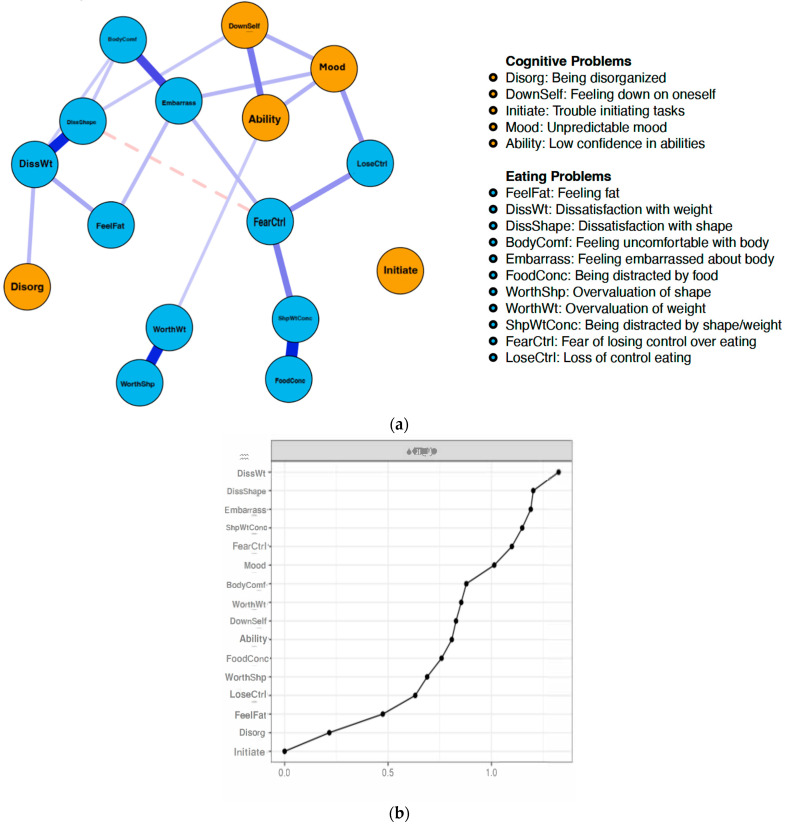
(**a**). Network structure of non-reporters of trauma. Blue circles (i.e., nodes) represent eating disorder symptoms (i.e., EDE-Q), and orange circles represent cognitive symptoms (i.e., CAARS). Thicker lines between nodes represent stronger associations. Dashed lines represent negative correlations. (**b**). Strength centrality plot of eating problems and cognitive problems in the network of non-reporters of trauma. Strength indices are standardized (*M* = 0, *SD* = ±1), where higher scores indicate greater centrality. DissWt = Dissatisfaction with weight; DissShape = Dissatisfaction with shape; Embarrass = Feeling embarrassed about body; ShpWtConc = Being distracted by shape/weight; FearCtrl = Fear of losing control over eating; Mood = Unpredictable mood; BodyComf = Feeling uncomfortable with body; WorthWt = Overvalutaion of weight; DownSelf = Feeling down on oneself; Ability = Low confidence in abilities; FoodConc = Being distracted by food; WorthShp = Overvaluation of shape; LoseCtrl = Loss of control eating; FeelFat = Feeling Fat; Disorg = Being disorganized; Initiate = Trouble initiating tasks.

Figure 2a,b depicts the network structure and strength centrality of the T− sample. The CP nodes with the greatest strength centrality among non-reporters of childhood trauma were unpredictable mood (*strength* = 1.11), feeling down on oneself (*strength* = 0.67), and having low confidence (*strength* = 0.63). The EP nodes with the greatest strength centrality in this network were dissatisfaction with weight (*strength* = 1.56), dissatisfaction with shape (*strength* = 1.45), and embarrassment seeing one’s body (*strength* = 1.40).

### 3.3. Network Comparison

Regarding the network comparison test (NCT), we were unable to test for specific differences in network structure invariance due to the relatively small sample sizes. We were, however, able to run a global strength invariance test, which is among the options for comparing networks when using the NCT package in R. The global strength invariance test indicates whether the sum of the absolute values of all edges in the network varies significantly between each network. We did not find a significant difference (test statistic *S*: 0.83; *p* = 0.17) between the two networks, suggesting that the strength of relationships within our networks was more similar than it was dissimilar.

## 4. Discussion

Despite the importance of understanding cognitive disruptions and ED behaviors in the context of traumatic experiences, this was the first study to examine the empirical network structure of these relationships in a community sample. Our findings are in line with the CBT model of ED psychopathology [16] and also present several novel considerations.

As expected, overvaluation of weight/shape (i.e., the extent to which weight/shape influences how one thinks about oneself as a person) emerged with high centrality in both networks but was more salient to the T+ network compared to the T− network. This finding is supported by prior literature, which suggests that traumatic experiences may be linked to eating disorder symptoms via weight/shape overvaluation in structural equation models [41]. Further, it is suggested that the specific effects of trauma on cognition (i.e., negative alterations in cognition) are associated with increased weight/shape valuation and global ED symptoms [42].

Additionally, the T+ network showed a connectedness between task initiation difficulties (CP) and being distracted by food/eating (EP)—a relationship that did not emerge among non-reporters of trauma. This finding supports the possible role of cognition as an important pathway involved in trauma and eating pathology. There is an emerging body of research suggesting that attentional bias for food-related information presents in ED psychopathology (e.g., [43]). These attentional biases have also been studied in post-traumatic stress symptoms [44], which suggests that executive functioning deficits are not uncommon among trauma-exposed individuals [45]. According to this research, trauma experienced during early development can disrupt the hypothalamic-pituitary-adrenal (HPA) axis and associated regions (i.e., prefrontal cortex), which are responsible for maintaining attention and planning, initiating and organizing tasks [46,47]. Our study is the first, however, to demonstrate a direct interaction, via a network approach, between eating-related attentional bias and cognitively driven mechanisms (i.e., task initiation) in the context of childhood trauma.

Alternatively, among non-reporters of childhood trauma, dissatisfaction with weight and shape were among the most central symptoms. The finding that dissatisfaction, but less so overvaluation, plays an important role in this network suggests that body image concerns are prevalent irrespective of trauma exposure status, but that concerns may differ according to trauma history. Research has examined these relative contributions of dissatisfaction versus overvaluation to clinical outcomes in eating disorders. Findings from these prior studies suggest that dissatisfaction, while often overlooked in comparison to overvaluation, is an important predictor of disordered eating across the age spectrum, although it is generally regarded as being more “normative” than overvaluation, especially among women [48,49,50]. Additionally, it has been demonstrated that body dissatisfaction is most consistently associated with depressive symptoms [50], which is further supported by our finding of mood symptoms being central in the context of the T- network.

Our findings demonstrate support for our hypothesis that a sense of self would be relevant to both networks (although these symptoms were overall more central among non-reporters of childhood trauma). While this construct represents a secondary feature of CPs in our study, findings suggest that the putative downstream consequences of cognitive difficulties on self-concept may have implications for eating-related disturbances. Moreover, negative self-concept in the context of cognitive difficulties may be a pathway through which such deficits are linked to eating-related problems, although more research is needed to understand this relationship.

Altogether, our results demonstrate that cognitively involved EPs and CPs were implicated in our T+ network, while affective symptoms were salient to our T− network (i.e., *unpredictable mood*). Moreover, while primary cognitive deficits (i.e., *task initiation difficulties*) presented in our T+ network, the proposed consequences of these deficits (i.e., *poor self-concept*) presented in our T− network. Notwithstanding these features, our network comparison test did not reveal statistically significant differences between reporters and non-reporters of trauma. It is possible, however, that differences may have been detected according to structure had our analyses been sufficiently powered to conduct these additional analyses. Both networks demonstrated the relative importance of body image concerns (i.e., overvaluation of weight/shape, dissatisfaction with weight/shape), which is in line with transdiagnostic models of eating disorders. It is important, however, that these relationships be examined across age, especially given that symptom networks can differ across developmental periods [51]. Additionally, these relationships should be studied in the context of types and severity of traumatic exposure and among clinical and non-clinical groups of women from diverse racial and ethnic backgrounds. Regarding trauma exposure specifically, there is literature to suggest that among those who have experienced sexual violence, ED psychopathology is more likely to develop relative to other forms of trauma [52].

Our findings have clinical implications, suggesting that irrespective of trauma status, body image concerns are an important intervention target in treating eating-related psychopathology. Among those reporting trauma exposure, it is worthwhile to consider executive control processes involved in maladaptive eating as an additional treatment target. Future research would benefit from examining the effectiveness of executive functioning interventions—i.e., cognitive remediation therapy (CRT; [53]) on alleviating comorbid ED psychopathology and post-traumatic stress symptoms. Moreover, our study contributes to a small but growing body of literature suggesting that cognitive deficits characteristic of ADHD may have impacts beyond their cardinal symptoms by influencing one’s sense of self and perceived abilities.

There are several limitations of our study that may inform the next steps. First, we have a relatively small sample size given the recommendations of having at least three participants per parameter [54], and we did not collect information on psychological/psychopharmacological interventions that our participants were engaged in. Future research should replicate our results with a larger sample and should explore how engagement with empirically based interventions contributes to network dynamics. Second, community research may limit generalizability to a clinical population; however, community samples are often overlooked in scientific research, and this study points to the relevance of eating pathology and cognitive presentation in the context of trauma exposure among these populations. Future research should explore our relationships of interest in a clinical sample to determine if the strength of these relationships and most central symptoms vary depending on the extent of psychopathology (i.e., sub-threshold vs. threshold). Finally, while our determination of what constitutes a traumatic event follows the diagnostic criterion outlined in the DSM-IV, prior research suggests that the expression of psychopathological symptoms can vary widely depending on the nature of the traumatic event [55]. As such, future research may benefit from examining ED psychopathology and cognitive symptoms according to formal diagnostic criteria for PTSD and stratifying our analyses according to the nature of the traumatic event experienced.

## 5. Conclusions

The present study is, to our knowledge, the first to use network analysis to examine the interrelatedness between cognitive deficits (and their suggested impacts on the self) and ED psychopathology—and how early trauma exposure influences these factors. Our findings demonstrate that while networks shared core features (i.e., body image concerns), cognitive symptoms may be more relevant to EP/CP relationships for those who have experienced trauma, while affective symptoms and more “normative” eating-related concerns (i.e., body dissatisfaction) demonstrate relatively stronger impacts in the absence of trauma exposure. Our findings have implications for ED assessment and the development of evidence-based interventions, including the identification of possible treatment targets to inform just-in-time interventions.

## Figures and Tables

**Table 1:** Descriptive statistics for the final sample of reporters and non-reporters of childhood trauma (*n* = 217).

	**T+ Sample (*n* = 116)**	**T− Sample (*n* = 101)**
**Mean Age In Years (SD)**	48 (17.4)	46 (15.7)
**Mean Education In Years (SD)**	15.5 (2.2)	15.6 (2.5)
**% Female**	68.5%	65.4%
**% Race**		
American Indian/Native Alaskan	<1 (*n* = 1)	<1 (*n* = 1)
Asian	3.4 (*n* = 4)	1.72 (*n* = 2)
Black or African American	6.03 (*n* = 7)	11.8 (*n* = 12)
Native Hawaiian/Other Pacific Islander	<1 (*n* = 1)	<1 (*n* = 1)
White	86.2 (*n* = 100)	83.1 (*n* = 84)
Other	2.6 (*n* = 3)	<1 (*n* = 1)

## Data Availability

The datasets presented in this article are not readily available because they are protected by a data use agreement. Requests to access the datasets should be directed to the NKI-RS.

## Supplementary Materials

The following supporting information can be downloaded at: https://www.mdpi.com/article/10.3390/healthcare13060630/s1. Figure S1: Bootstrapped centrality scores with case dropping for the T+ network; Figure S2: Bootstrapped centrality scores with case dropping for the T− network. Table S1: Abbreviations and corresponding items for EP nodes (i.e., EDE-Q); Table S2: Abbreviations and corresponding items for CP nodes (i.e., CAARS-S:S).

## Abbreviations

The following abbreviations are used in this manuscript:

ED	eating disorder
ADHD	attention-deficit/hyperactivity disorder
PTSD	post-traumatic stress disorder
NKI-RS	Nathan Kline Institute Rockland Sample
EP	eating problem
CP	cognitive problem
