# Supplementary material for: Childhood Trauma, Cognition, and Eating Psychopathology: A Network Analysis
Source: Healthcare (Basel). 2025 Mar 14;13(6):630. doi: 10.3390/healthcare13060630 (PMC11941857; doi:10.3390/healthcare13060630)
Supplement: Supplementary file 1 [file healthcare-13-00630-s001.zip › healthcare-3490795-supplementary.pdf]

Supplementary Materials

Table S1. Abbreviations and corresponding items for EP nodes (i.e., EDE-Q)

Node Label / Abbreviation	EDE-Q Item
BodyComf	How uncomfortable have you felt seeing your body (for example, seeing your shape in the mirror, in a shop window reflection, while undressing or taking a bath or shower)?
DissShape	How dissatisfied have you been with your shape?
DissWt	How dissatisfied have you been with your weight?
FoodConc	Has thinking about food, eating, or calories made it very difficult to concentrate on things you are interested in (for example, working, following a conversation, or reading)?
ShpWtConc	Has thinking about shape or weight made it very difficult to concentrate on things you are interested in (for example, working, following a conversation, or reading)?
Embarrass	How uncomfortable have you felt about others seeing your shape or figure (for example, in communal changing rooms, when swimming, or wearing tight clothes)?
FearCtrl	Have you had a definite fear of losing control over eating?
FeelFat	Have you felt fat?
LoseCtrl	On how many of these times did you have a sense of having lost control over your eating (at the time that you were eating)?
WorthShp	Has your shape influenced how you think about (judge) yourself as a person?
WorthWt	Has your weight influenced how you think about (judge) yourself as a person?

Table S2. Abbreviations and corresponding items for CP nodes (i.e., CAARS-S:S)

Node Label / Abbreviation	CAARS Item
Disorg	I am disorganized.
DownSelf	I get down on myself.
Ability	I wish I had greater confidence in my abilities.
Initiate	I have trouble getting started on a task.
Mood	My moods are unpredictable.

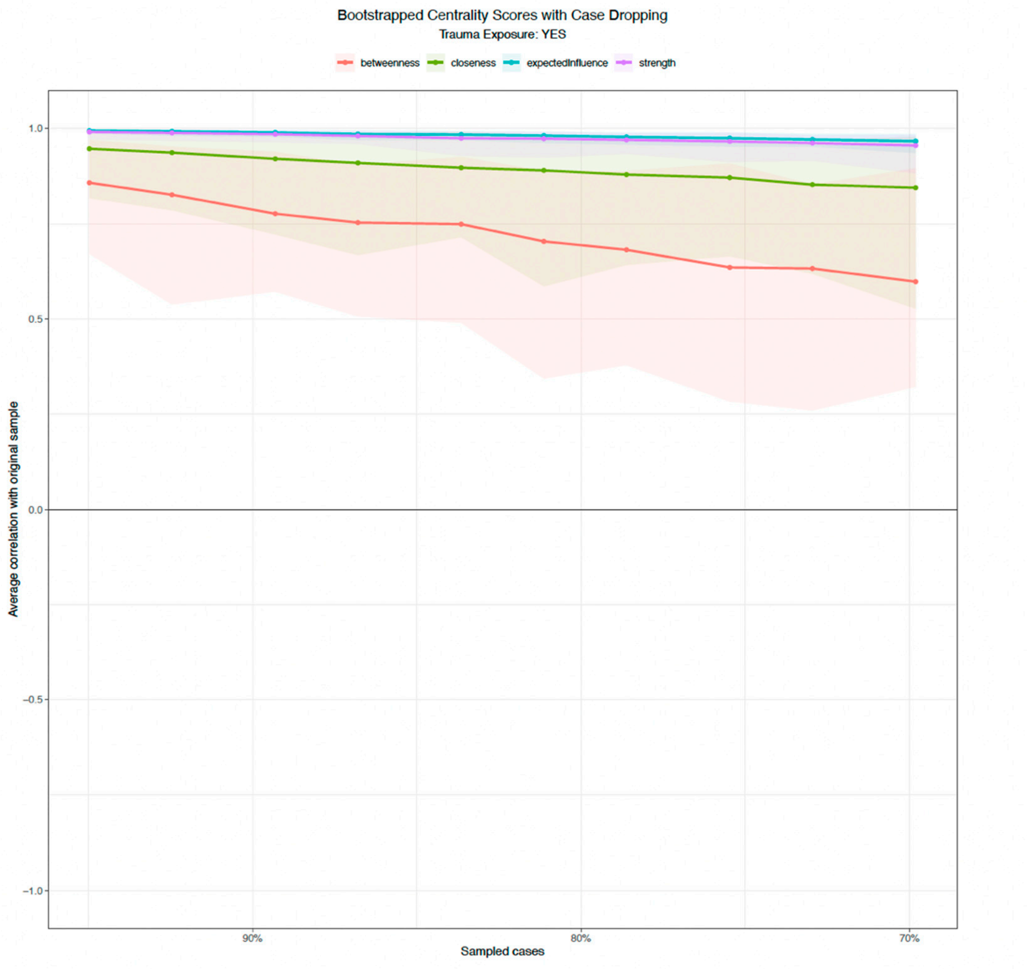

\

Figure S1. Bootstrapped centrality scores with case dropping for the T+ network.

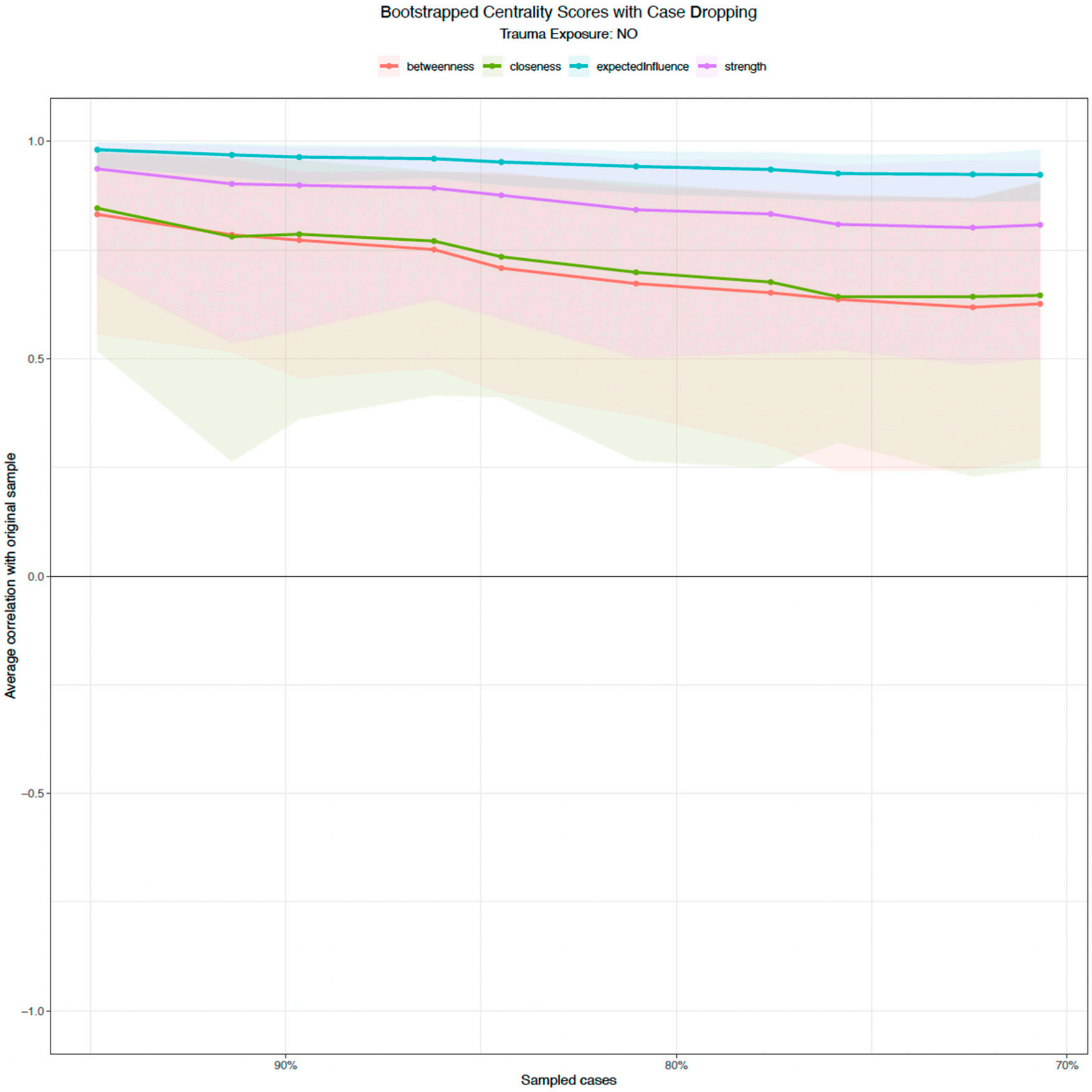

Figure S2. Bootstrapped centrality scores with case dropping for the T- network.
